# Acclimatory responses of *Arabidopsis* to fluctuating light environment: comparison of different sunfleck regimes and accessions

**DOI:** 10.1007/s11120-012-9757-2

**Published:** 2012-06-24

**Authors:** Philipp Alter, Anne Dreissen, Fang-Li Luo, Shizue Matsubara

**Affiliations:** 1 IBG-2: Pflanzenwissenschaften, Forschungszentrum Jülich, 52425 Jülich, Germany; 2 Aachen University of Applied Sciences, 52066 Aachen, Germany; 3 Present Address: Cell Biology and Plant Biochemistry, Universität Regensburg, 93053 Regensburg, Germany; 4 College of Nature Conservation, Beijing Forestry University, Beijing, 100083 China

**Keywords:** Acclimation, *Arabidopsis thaliana*, Non-photochemical quenching, Photoprotection, Sunflecks, Xanthophyll cycle

## Abstract

Acclimation to fluctuating light environment with short (lasting 20 s, at 650 or 1,250 μmol photons m^−2^ s^−1^, every 6 or 12 min) or long (for 40 min at 650 μmol photons m^−2^ s^−1^, once a day at midday) sunflecks was studied in *Arabidopsis thaliana*. The sunfleck treatments were applied in the background daytime light intensity of 50 μmol photons m^−2^ s^−1^. In order to distinguish the effects of sunflecks from those of increased daily irradiance, constant light treatments at 85 and 120 μmol photons m^−2^ s^−1^, which gave the same photosynthetically active radiation (PAR) per day as the different sunfleck treatments, were also included in the experiments. The increased daily total PAR in the two higher constant light treatments enhanced photosystem II electron transport and starch accumulation in mature leaves and promoted expansion of young leaves in Columbia-0 plants during the 7-day treatments. Compared to the plants remaining under 50 μmol photons m^−2^ s^−1^, application of long sunflecks caused upregulation of electron transport without affecting carbon gain in the form of starch accumulation and leaf growth or the capacity of non-photochemical quenching (NPQ). Mature leaves showed marked enhancement of the NPQ capacity under the conditions with short sunflecks, which preceded recovery and upregulation of electron transport, demonstrating the initial priority of photoprotection. The distinct acclimatory responses to constant PAR, long sunflecks, and different combinations of short sunflecks are consistent with acclimatory adjustment of the processes in photoprotection and carbon gain, depending on the duration, frequency, and intensity of light fluctuations. While the responses of leaf expansion to short sunflecks differed among the seven *Arabidopsis* accessions examined, all plants showed NPQ upregulation, suggesting limited ability of this species to utilize short sunflecks. The increase in the NPQ capacity was accompanied by reduced chlorophyll contents, higher levels of the xanthophyll-cycle pigments, faster light-induced de-epoxidation of violaxanthin to zeaxanthin and antheraxanthin, increased amounts of PsbS protein, as well as enhanced activity of superoxide dismutase. These acclimatory mechanisms, involving reorganization of pigment–protein complexes and upregulation of other photoprotective reactions, are probably essential for *Arabidopsis* plants to cope with photo-oxidative stress induced by short sunflecks without suffering from severe photoinhibition and lipid peroxidation.

## Introduction

Light in natural environments is highly variable in both intensity and spectral composition. Pronounced temporal fluctuations and spatial heterogeneity also characterize the dynamic nature of light environment. For many plants, to rely on this energy source for life means to deal with its regular and irregular changes. Irregular changes in light environment occur in various ways, but the most common causes include variation in weather and cloud movement, development and destruction of leaves, branches, or canopy, and fluttering of leaves by wind. Some changes are long-lasting, such as gap formation in forest canopies which allows more sunlight to reach the forest floor. Short-term fluctuation of light occurs in forest understorey or inside dense crop canopies. In both cases, rays of sunlight penetrate the canopy in the form of “sunflecks” to expose shade-grown leaves and plants to bursts of high light (HL). On clear days, sunflecks account for 20~80 % of photosynthetically active radiation (PAR) available for understorey plants growing in different types of forests, or 40~90 % within soybean canopies (Pearcy 1990 and references therein). Hence, sunfleck utilization efficiency, e.g., due to photosynthetic induction and induction loss (Chazdon and Pearcy 1986a; Pons et al. 1992), has been of ecological and agricultural interest.

Responses to sunflecks vary among species or even within a species depending on the duration, frequency, and intensity of sunflecks (Chazdon and Pearcy 1986b; Sims and Pearcy 1993; Watling et al. 1997a; Yin and Johnson 2000; Leakey et al. 2004). When the sunfleck intensity is higher than what can be utilized in a given photosynthetic induction state, excessive light energy can lead to the formation of reactive oxygen species (ROS) and photo-oxidative stress, and hence can trigger photoprotective reactions in plants, such as thermal energy dissipation commonly measured as non-photochemical quenching (NPQ) of chlorophyll (Chl) *a* fluorescence. Sunflecks can thus become a source of energy and carbon gain (i.e., photosynthesis and growth), as well as photodamage for leaves and plants growing in low light (LL). However, most of the previous studies were conducted by focusing on either photosynthetic or photoprotective responses to sunflecks (e.g. Pearcy and Calkin 1983; Chazdon and Pearcy 1986a,b; Pons et al. 1992; Sims and Pearcy 1993; Ögren and Sundin 1996; Watling et al. 1997a; Yin and Johnson 2000; Krause et al. 2001; Leakey et al. 2004). Few studies, in which both responses were simultaneously analyzed in plants growing in the field (Logan et al. 1997; Watling et al. 1997b; Adams et al. 1999), showed adjustment of the partitioning of absorbed light energy between photochemistry and photoprotection of photosystem II (PSII) in response to dynamically changing PAR over a day, somewhat increased accumulation of the xanthophyll-cycle pigments (violaxanthin, V; antheraxanthin, A; zeaxanthin, Z), and retention of A and Z in leaves after exposure to strong sunflecks. The light-induced de-epoxidation of V to A and Z in the xanthophyll cycle is known to be involved in photoprotective thermal energy dissipation (Demmig-Adams 1990; Niyogi et al. 1998) and protection of thylakoid membranes against lipid peroxidation (Havaux and Niyogi 1999; Havaux et al. 2007). Thus, upregulation of these photoprotective mechanisms seems to be crucial for acclimation of LL-grown plants to fluctuating light environment with sunflecks.

Compared to diurnal changes in photosynthesis and photoprotection under fluctuating light environment or physiological and biochemical properties of leaves acclimated to sunfleck conditions, much less is known about the acclimatory processes which bring about such alterations in leaf properties. How quickly can the capacities of photoprotection and carbon gain change in leaves during acclimation to sunfleck conditions? Are the acclimatory processes for photosynthesis and photoprotection similarly or differently affected by duration, frequency and intensity of sunflecks? In order to address these questions, we exposed LL-grown plants of the model species *Arabidopsis thaliana* (hereafter *Arabidopsis*), a common laboratory accession Columbia-0 (Col-0), to well-defined sunfleck conditions in a controlled climate chamber and monitored acclimatory changes in PSII activities, starch accumulation, and leaf growth for 7 days. Owing to the availability of large genetic resources and extensive knowledge accumulating at all levels from genes to whole plant, *Arabidopsis* has become an important model system in plant biology. Unlike forest understorey plants, however, *Arabidopsis* usually occupies open or disturbed habitats and is a poor competitor in dense vegetations (Koornneef et al. 2004). This may imply limited capacities of *Arabidopsis* plants to grow under LL + sunflecks environments, possibly due to low carbon gain and/or insufficient photoprotection in such conditions.

Effects of sunfleck duration, frequency, and intensity on the acclimatory responses were examined by applying short sunflecks (SSF, lasting 20 s) at two different intensities (650 or 1,250 μmol photons m^−2^ s^−1^) and two different intervals (every 6 or 12 min) or long sunflecks (LSF, lasting 40 min) at 650 μmol photons m^−2^ s^−1^ once a day. The sunfleck treatments were performed under PAR of the LL growth condition (50 μmol photons m^−2^ s^−1^). To distinguish the effects of sunflecks from those of different daily total PAR, constant light of moderately higher intensities (85 and 120 μmol photons m^−2^ s^−1^) were also included in the experiment; these treatments gave the same daily total PAR as the different sunfleck treatments.

After having found pronounced SSF-induced upregulation of NPQ in mature leaves of Col-0, the accession for which limited HL acclimation of the photosynthetic capacity has been reported (Athanasiou et al. 2010), we asked whether this type of acclimatory response to SSF is common among different *Arabidopsis* accessions. Native habitats of *Arabidopsis* are Europe and Central Asia, but it has been spread in many places across the latitudinal range between North Scandinavia and mountains of Tanzania and Kenya (Koornneef et al. 2004). A second series of experiments was conducted by monitoring SSF-induced responses of NPQ and leaf expansion in seven accessions from various geographic origins. Finally, biochemical traits associated with tropical rainforest species in sunfleck environments (Logan et al. 1997; Watling et al. 1997b; Adams et al. 1999) or *Arabidopsis* plants acclimated to constantly HL or photo-oxidative stress (Abarca et al. 2001; Ballottari et al. 2007; Kalituho et al. 2007) were ascertained by measuring photosynthetic pigment composition, the level of PsbS protein, and superoxide dismutase (SOD) activity in three accessions showing contrasting responses of leaf expansion to sunflecks.

The results show distinct effects of constant PAR, LSF, and SSF on acclimation of Col-0 plants and highlight strong photoprotective responses to SSF that are conserved in different *Arabidopsis* accessions.

## Materials and methods

### Plant materials and growth conditions

Seeds of *Arabidopsis thaliana* (L.) Heynh. were sown in small germination trays (13 × 17 × 5 cm) containing soil (type VM; Balster Einheitserdewerk, Fröndenberg, Germany). In the first experiment of light regime comparison, germination trays with seeds of the common laboratory strain Col-0 were placed for 2 weeks under PAR of ca. 80 μmol photons m^−2^ s^−1^ provided by fluorescent lamps (Fluora L36 W/77; Osram, Munich, Germany) with a photoperiod of 12 h/12 h (day/night) and 23 °C/18 °C air temperature at constant 60 % relative air humidity. In the second experiment to compare accessions, six additional accessions were included along with Col-0: C24 (Coimbra, Portugal), Eri (Eringsboda, Sweden), L*er* (*erecta* line isolated from the irradiated Laibach Landsberg population originating from Gorsow Wipolski, Poland), Kyo (Kyoto, Japan), An-1 (Antwerp, Belgium), and Cvi (Cape Verde Island). Seeds of these accessions were kindly provided by Maarten Koornneef (Max Planck Institute for Plant Breeding Research, Cologne). In the second experiment, seeds were stratified at 8 °C in the dark for 4 days before transferring to the condition described above.

After 2 weeks in 80 μmol photons m^−2^ s^−1^, seedlings were transferred from the germination trays to pots (7 × 7 × 8 cm; one plant per pot) filled with soil (type ED73; Balster Einheitserdewerk), and PAR was reduced to 50 μmol photons m^−2^ s^−1^ without changing other conditions in the climate chamber. Experiments for leaf growth and carbohydrate analysis were started after 2 weeks of cultivation under 50 μmol photons m^−2^ s^−1^; other experiments were started a week later, i.e., after 3 weeks of cultivation under 50 μmol photons m^−2^ s^−1^. Plants were watered daily or every other day throughout the cultivation and experiments.

### Light regimes

In the first experiment, plants were exposed to different light regimes for 7 days without changing the other conditions in the climate chamber: constant daytime PAR of 50 μmol photons m^−2^ s^−1^ (C 50), “long sunflecks” (LSF, lasting 40 min) of 650 μmol photons m^−2^ s^−1^ once a day at around midday (LSF 650), “short sunflecks” (SSF, lasting 20 s) of 650 μmol photons m^−2^ s^−1^ every 6 min during the daytime (SSF 650/6), and SSF of 1,250 μmol photons m^−2^ s^−1^ every 12 (SSF 1250/12) or 6 min (SSF 1250/6) during the daytime. All sunfleck treatments were performed under the C 50 condition during the day. Additionally, some plants were transferred to constant daytime PAR of ca. 85 (C 85) or 120 (C 120) μmol photons m^−2^ s^−1^; the daily total PAR in these treatments was comparable with the values in the sunfleck treatments (ca. 3.6 mol photons m^−2^ day^−1^ in C 85, LSF 650, SSF 650/6 and SSF 1250/12; ca. 5.1 mol photons m^−2^ day^−1^ in C 120 and SSF 1250/6). The daily total PAR in C 50 was ca. 2.1 mol photons m^−2^ day^−1^. Light intensity was measured in a horizontal position at the height of the plants using a PAR meter (LI-250A; LI-COR, Lincoln, NE, USA).

Constant illumination (C 50, C 85, and C 120) was provided by fluorescent lamps (Fluora L36 W/77; Osram). Long sunflecks (LSF 650) were applied by placing plants under mercury-arc lamps (GW 84 463; GEWISS, Merenberg, Germany) installed in the same climate chamber. Treatments with short sunflecks (SSF 650/6, SSF 1250/12 and SSF 1250/6) were performed using halogen spotlight lamps (Haloline; Osram) aligned in a row. We note that these light sources had different spectral compositions, which could have had additional effects on plants. Under constant illumination (C 50, C 85 and C 120), leaf temperature was around 21~22 °C in the light, whereas it increased in the SSF conditions to reach 23~24 °C in the afternoon. The LSF raised the leaf temperature up to 27~28 °C during the 40-min treatment.

A computer-assisted setup was built to control the duration and frequency of SSF. The halogen lamps were turned on shortly before each sunfleck event and moved over the plants in one direction (like a scanner); the velocity of the lamps’ movement was chosen such that each plant was exposed to the halogen spotlight for ca. 20 s. Upon reaching the end position, the lamps were turned off and brought back to the start position to wait until the next event. This program was repeated every 6 (SSF650/6 and SSF 1250/6) or 12 min (SSF 1250/12) during the 12-h day period. The intensity of sunflecks was modified by changing the halogen lamps (120 or 500 W) and adjusting the distance between lamps and plants.

Only the treatments of C 50 and SSF 1250/6 were used for comparison of different accessions in the second experiment.

### Chlorophyll *a* fluorescence analysis

Chlorophyll *a* fluorescence was measured in the morning using a PAM 2100 (Walz, Effeltrich, Germany). Only mature leaves, which had existed before starting the experiments, were used for measurements. Plants were transferred from the climate chamber to the laboratory at the end of the night period and kept in the dark until measurements. Following the measurement of the maximal PSII efficiency (*F*_v_/*F*_m_) in a dark-adapted state, actinic light (ca. 1,000 μmol photons m^−2^ s^−1^) was applied for 8 (in the first experiment) or 5 min (in the second experiment) by the built-in white halogen lamp of PAM 2100. Non-photochemical fluorescence quenching, the reduction state of the bound primary quinone Q_A_ in PSII (1-qp), and the effective PSII efficiency (Δ*F*/$F_{\text{m}}^{\prime }$) were determined in illuminated leaves. In the first experiment with different light regimes; dark relaxation of NPQ was also monitored for 14 min after switching off the actinic light.

The fluorescence parameters were calculated as follows:

1 $$ F_{\text{v}} /F_{\text{m}} = \;(F_{\text{m}} - F_{0} )/F_{\text{m}} , $$

2 $$ {\text{NPQ}} = (F_{\text{m}} - F_{\text{m}}^{\prime } )/F_{\text{m}}^{\prime } , $$

3 $$ {\text{qp}} = (F_{\text{m}} - F)/(F_{\text{m}}^{\prime } - F_{0}^{\prime } ), $$

4 $$ \Updelta F/F_{\text{m}}^{\prime } = (F_{\text{m}} - F)/F_{\text{m}}^{\prime } , $$

where *F*_m_ and *F*_o_ are the maximal and minimal fluorescence intensity in dark-adapted leaves and $F_{\text{m}}^{\prime }$, $F_{ 0}^{\prime }$ and *F* are the maximal, minimal and actual fluorescence intensity in light-adapted leaves, respectively. For fluorescence nomenclature, see Schreiber (2004). Relative electron transport rate of PSII (ETR) was calculated according to the following equation:

5 $$ {\text{ETR}} = 0.84 \times 0.5 \times {\text{PAR}} \times \Updelta F/F_{m}^{\prime } $$

assuming 84 % absorptance of the incident PAR by leaves and equal turnover of PSII and PSI (Schreiber 2004) in all treatments.

### Leaf growth analysis

The projected total leaf area was measured for each plant early in the afternoon every other day using the GROWSCREEN (in the first experiment; Walter et al. 2007) or GROWSCREEN FLUORO system (in the second experiment; Jansen et al. 2009). At this time of the day, leaves of *Arabidopsis* plants are positioned almost horizontally above the soil in all light regimes used in the present study.

Data of the projected total leaf area were fitted to an exponential growth function for each treatment and accession:

6 $$ A_{x} = A_{0} \times \exp^{(b \times x)} , $$

where *A*_*x*_ and *A*_0_ are the projected total leaf area on day *x* and day 0, respectively, and *b* is the growth factor. The relative growth rate (RGR,  % day^−1^) of the projected total leaf area was obtained by multiplying *b* by 100.

### Carbohydrate assay

Leaf samples for carbohydrate assay were harvested after 10 h of illumination by different light regimes on the second and fifth day of the treatments. As described for the Chl fluorescence analysis, only mature leaves, which had existed before starting the experiments, were used for the analysis. After excision, leaves were quickly weighed, frozen in liquid N_2_, and stored at −80 °C until extraction. Soluble sugars (glucose, fructose and sucrose) and starch were extracted from the leaves as described by Czech et al. (2009). Concentrations of soluble sugars were determined according to Jones et al. (1977). Starch concentration was measured as glucose after enzymatic digestion with α-amylase and amyloglucosidase (Czech et al. 2009). Carbohydrate contents were expressed relative to leaf fresh weight (μmol g^−1^ FW).

### Analysis of photosynthetic pigments

Leaf disks (0.77 cm^2^) were taken from mature leaves early in the morning on day 0 (before the treatments) and on day 7 (after 7 days under different light regimes) to analyze photosynthetic pigments. The mature leaves used for sampling on day 7 were those that existed already on day 0. Two samples were collected from each plant: a “dark” sample taken at the end of the night period and a “light” sample taken after exposure of plants to halogen lamps (Haloline; Osram) of ca. 1,000 μmol photons m^−2^ s^−1^ for 5 min. The latter condition is comparable with the actinic illumination used for NPQ measurements in the second experiment. Leaf disks were immediately frozen in liquid N_2_ and stored at −80 °C until pigment extraction.

Photosynthetic pigments were extracted by grinding frozen leaf disks in 1 mL acetone. The homogenate was then centrifuged at 13,000 rpm for 5 min and filtered (0.45-μm True Syringe Filter; Alltech Associates) before injection (20 μL) into the HPLC system. Chlorophylls and carotenoids were separated with an Allsphere ODS-1 column (5 μm, 250 × 4.6 mm; Alltech Associates) at a constant flow rate of 1 mL min^−1^ according to the method modified from Gilmore and Yamamoto (1991). Pigments were detected using a Waters 996 photodiode array detector (Waters Corporation) and the peak area of chromatograms was integrated at 440 nm with the Empower software (Waters Corporation).

### Western blot analysis

Leaf samples for PsbS protein analysis were taken early in the morning on day 0 and day 7 in parallel with the “dark” samples of pigment analysis. The leaves were frozen in liquid N_2_ and stored at −80 °C. Proteins were extracted by homogenizing frozen leaves in a strongly denaturing buffer (7 M urea, 5 % SDS, 50 mM Tris–HCl (pH 7.6), and 5 % β-mercaptoethanol) followed by centrifugation at 13,000 rpm for 10 min at 4 °C. Samples from three replicate plants were pooled together for each treatment and accession. Protein samples containing 2 μg Chl were loaded on 14 % Tris–glycine–SDS gels. After electrophoresis, proteins were transferred to nylon membranes (Roche Diagnostics) and blots were blocked with 8 % low-fat milk powder in TBS buffer (pH 7.6) for 1 h at room temperature before adding anti-PsbS antiserum (Bonente et al. 2008, kindly provided by Roberto Bassi, University of Verona, Verona, Italy). Blots were incubated in this buffer containing the anti-PsbS antiserum at room temperature under constant agitation overnight. The PsbS protein was detected through the reaction of alkaline phosphatase conjugated to the secondary antibody (Anti-Rabbit IgG; Sigma-Aldrich). The PsbS protein levels were evaluated using the AIDA Imaging Analyzer (raytest GmbH, Straubenhardt, Germany).

### Superoxide dismutase activity assay

Samples of mature leaves (as described for the pigment analysis) were harvested early in the morning on day 0 and day 7 to analyze SOD (EC 1·15·1·1) activity. Fresh weight of the leaves was quickly measured before freezing in liquid N_2_. Frozen leaves were homogenized in 3 mL of 50 mM sodium phosphate buffer (pH 7.8) at 4 °C. Following centrifugation at 4,000 rpm and 4 °C for 15 min, supernatant was collected and the SOD activity was determined by the method of Beyer and Fridovich (1987), which is based on the ability of SOD to inhibit reduction of nitro blue tetrazolium chloride by photochemically generated superoxide radicals. One unit of SOD activity was defined as the amount of enzyme needed for 50 % inhibition of the reduction rate measured at 560 nm. The values were normalized to the leaf FW (U g^−1^ FW).

### Malondialdehyde assay

In parallel with the analysis of SOD activity, concentration of malondialdehyde (MDA), a product of lipid peroxidation, was also measured in the same leaf extracts according to the protocol by Beligni and Lamattina (2002). Leaf extracts (0.6 mL) were mixed with 1 mL 0.6 % (w/v) thiobarbituric acid, heated to 95 °C for 20 min, and quickly cooled on ice. Then, the samples were centrifuged at 4,000 rpm and 4 °C for 15 min and absorption was measured in the supernatant at 532 nm. For background correction, absorption at 600 nm was subtracted from the value at 532 nm. Concentrations of MDA were calculated by the molar extinction coefficient of 1.56 × 10^5^ M^−1^ cm^−1^ and expressed relative to the leaf FW (nmol g^−1^ FW).

### Statistical test

Differences between treatments were statistically tested by Dunnett’s test of one-way ANOVA (between C 50 and other light regimes in the first experiment) or *t* test (between C 50 and SSF 1250/6 for each accession). For the second experiment, effects of accessions (Col-0, C24 and Eri) and treatments (C 50 and SSF 1250/6) were analyzed by two-way ANOVA. All statistical tests were performed by means of SigmaStat 2.0 (SPSS Inc., Chicago, IL, USA).

## Results

### Changes in PSII activity during acclimation to different sunfleck conditions

**Fig. 1:**
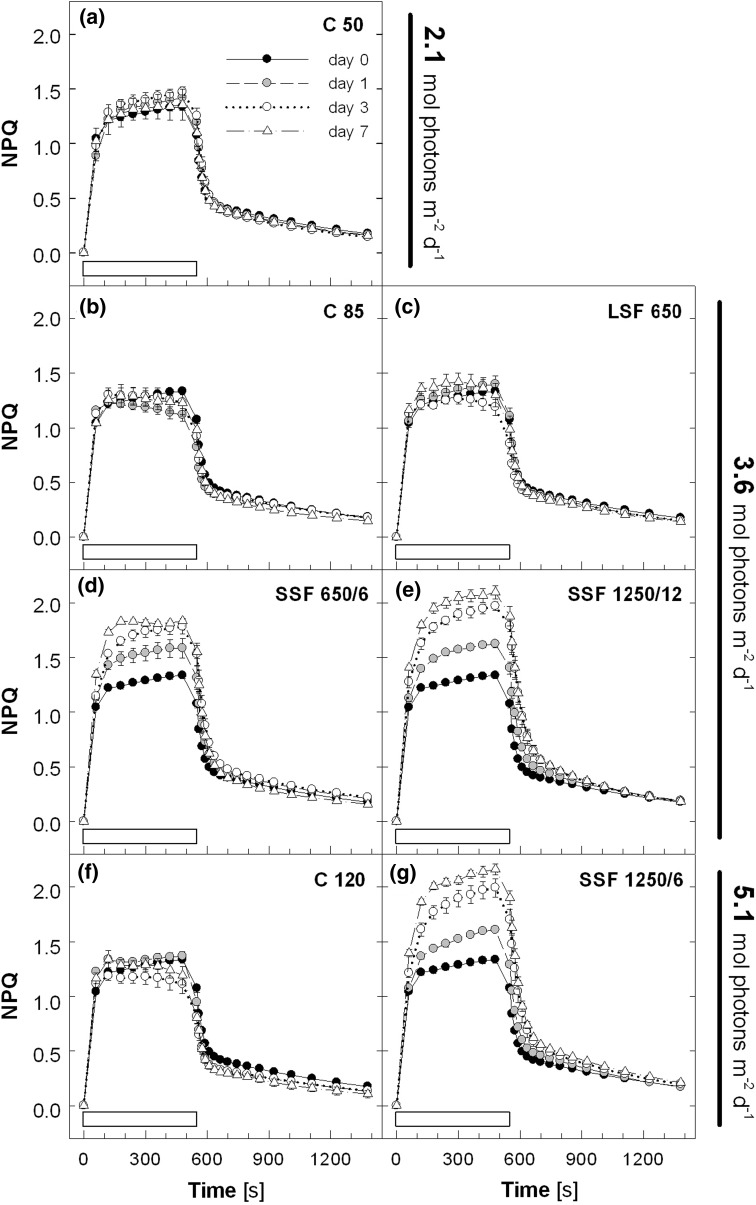
Non-photochemical quenching (NPQ) measured in leaves of Col-0 plants during 7-day exposure to different light regimes. NPQ was induced by illumination at 1,000 μmol photons m^−2^ s^−1^ (indicated by a *white bar* above the *x*-axis) for 8 min and dark relaxation was monitored subsequently for 14 min. The different light regimes in the climate chamber were: constant PAR of **a** ca. 50 (C 50), **b** 85 (C 85) and **f** 120 μmol photons m^−2^ s^−1^ (C 120) with a photoperiod of 12 h/12 h day/night; **c** long sunflecks of 650 μmol photons m^−2^ s^−1^ once a day at around midday (LSF 650); short sunflecks of **d** 650 μmol photons m^−2^ s^−1^ applied every 6 min (SSF 650/6), or 1,250 μmol photons m^−2^ s^−1^ every **e** 12 (SSF 1250/12) or **g** 6 min (SSF 1250/6). The treatments with LSF and SSF were performed under the C 50 condition. The daily total PAR was about **a** 2.1, **b**–**e** 3.6 or **f** and **g** 5.1 mol photons m^−2^ day^−1^. Plants were grown under C 50 and the light treatments were started on day 0. The maximal PSII efficiency of dark-adapted leaves (*F*_v_/*F*_m_) at the beginning of the measurements was 0.79~0.82 for all plants throughout the 7-day experiment. Data are means of five plants (±SE)

Acclimation of PSII activity to sunfleck conditions was studied in mature leaves of Col-0 plants by measuring Chl *a* fluorescence. Without significantly affecting the maximal PSII efficiency (*F*_v_/*F*_m_) in the dark (see legend to Fig. 1), different light regimes altered the maximal capacity of light-induced thermal energy dissipation determined as NPQ (Fig. 1). During the 7-day experiment, the plants in C 50 showed little change in the NPQ induction and relaxation patterns as well as the maximal NPQ level reached within 8 min of illumination at about 1,000 μmol photons m^−2^ s^−1^ (Fig. 1a). Transfer to C 85 (Fig. 1b) and C 120 (Fig. 1f) resulted in declining NPQ during the HL illumination, which, in the case of C 120, was accompanied by lower NPQ upon darkening. A similar tendency was found in LSF 650 although the changes were less obvious (Fig. 1c). The NPQ capacity increased in all plants transferred to the SSF conditions (Fig. 1d, e, g). The first sign of NPQ enhancement was seen in the SSF treatments within 24 h from the beginning of the experiments. The increase thereafter was more pronounced in SSF at higher PAR (SSF 1250/12 and SSF 1250/6); concomitantly, these plants retained higher NPQ during the dark relaxation period. At the end of the 14-min darkness, the lowest NPQ was found in C 120 (0.11) and the highest in SSF 1250/6 (0.21), which correspond to ca. 10 % and >17 % decrease, respectively, of the maximal fluorescence (*F*_m_) in the dark.

**Fig. 2:**
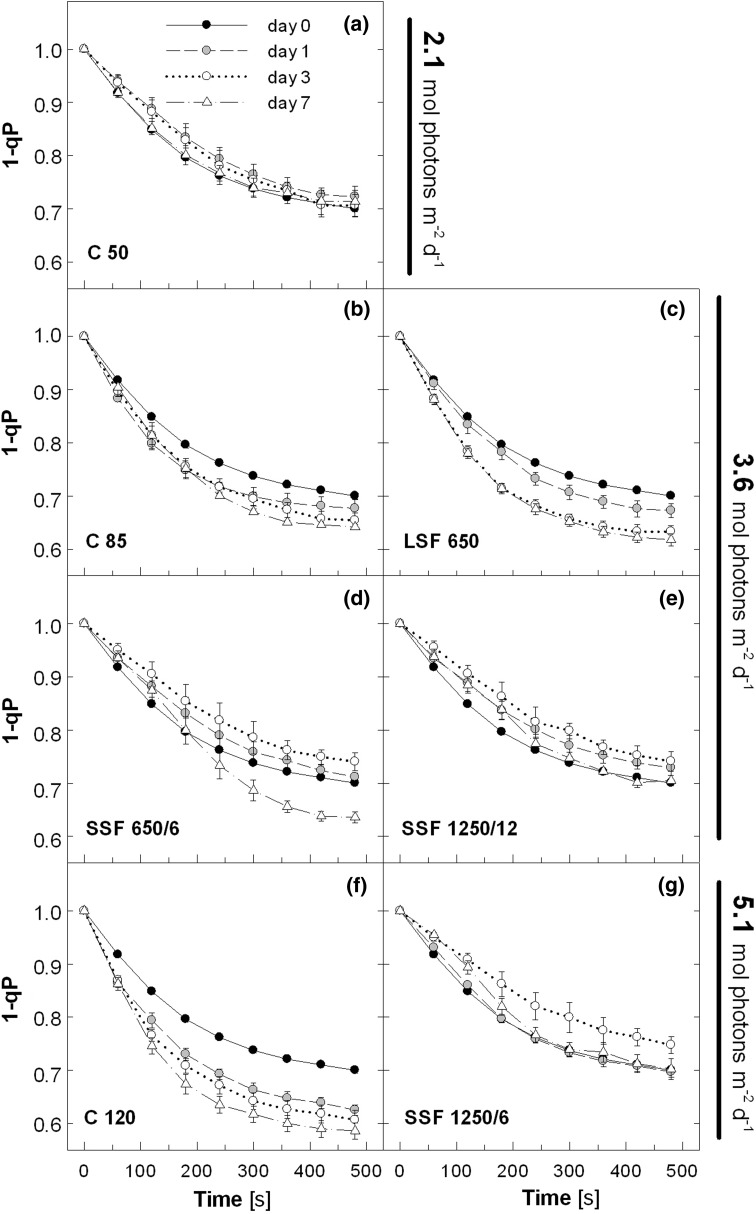
Reduction state of *Q*_A_ (1–qP) during light induction. The measurement protocol and the abbreviations of the light regimes are as described in the legend to Fig. 1. Data are means of five plants (±SE)

Distinct effects of the different light regimes were also evident in the *Q*_A_ reduction state of PSII estimated by the fluorescence parameter 1-qp (Fig. 2). The values of 1-qp decreased in the C 50 plants from 1 to around 0.7 during the HL illumination (Fig. 2a). Acclimation to constantly higher PAR in C 85 and C 120 enhanced Q_A_ oxidation, as indicated by lower 1-qp measured already on day 1 (Fig. 2b, f). Again, similar patterns were found in LSF 650 (Fig. 2c). In contrast, the plants treated with SSF first showed an increase in 1-qp as compared with day 0 (Fig. 2d, e, g), which was followed by a decrease (SSF 650/6; Fig. 2d) or return to the initial level (SSF 1250/12 and SSF 1250/6; Fig. 2e, g) by day 7. We note that the picture in Fig. 2 remained essentially the same when the Q_A_ reduction state was estimated by another parameter (1-ql; data not shown), which takes into account the connectivity among PSII complexes for light energy transfer (Kramer et al. 2004).

**Fig. 3:**
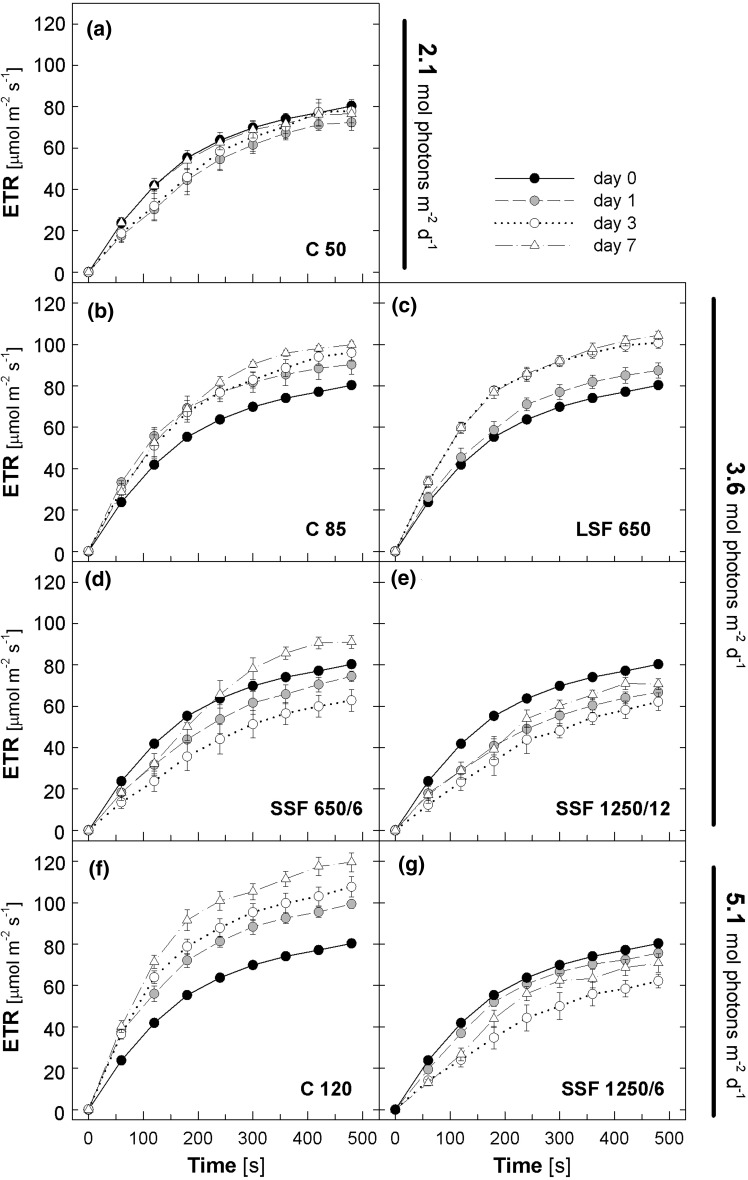
Electron transport rate (ETR) during light induction. The values were calculated from the effective PSII efficiency measured under 1,000 μmol photons m^−2^ s^−1^ as described in the legend to Fig. 1. Data are means of five plants (±SE)

Inverse patterns were found for ETR (Fig. 3), which is a proxy for the rate of electron transport at PSII. In the C 50 plants, ETR nearly reached saturation at around 80 μmol m^−2^ s^−1^ during 8-min illumination at 1,000 μmol photons m^−2^ s^−1^ (Fig. 3a). All plants that showed enhancement of Q_A_ oxidation during the 7-day acclimation (i.e., C 85, C 120, and LSF 650) also had increasing ETR; on day 7 the ETR values at the end of the illumination were ca. 100 μmol m^−2^ s^−1^ in C 85 and LSF 650 and 120 μmol m^−2^ s^−1^ in C 120 (Fig. 3b, c and f). Similarly, the increasing 1-qp detected in the SSF plants (Fig. 2d, e, g) was accompanied by decreasing ETR (Fig. 3d, e, g). The ETR values of these plants were the lowest on day 3 (ca. 60 μmol m^−2^ s^−1^), but recovered to 90 (SSF 650/6) or 70 μmol m^−2^ s^−1^ (SSF 1250/12 and SSF 1250/6) by day 7. It needs to be reminded, however, that the calculation of ETR based on constant light absorption and equal turnover of PSII and PSI (see “Materials and methods”) may not be uniformly applicable to plants undergoing acclimation to different light regimes.

### Carbohydrate accumulation under different sunfleck conditions

**Fig. 4:**
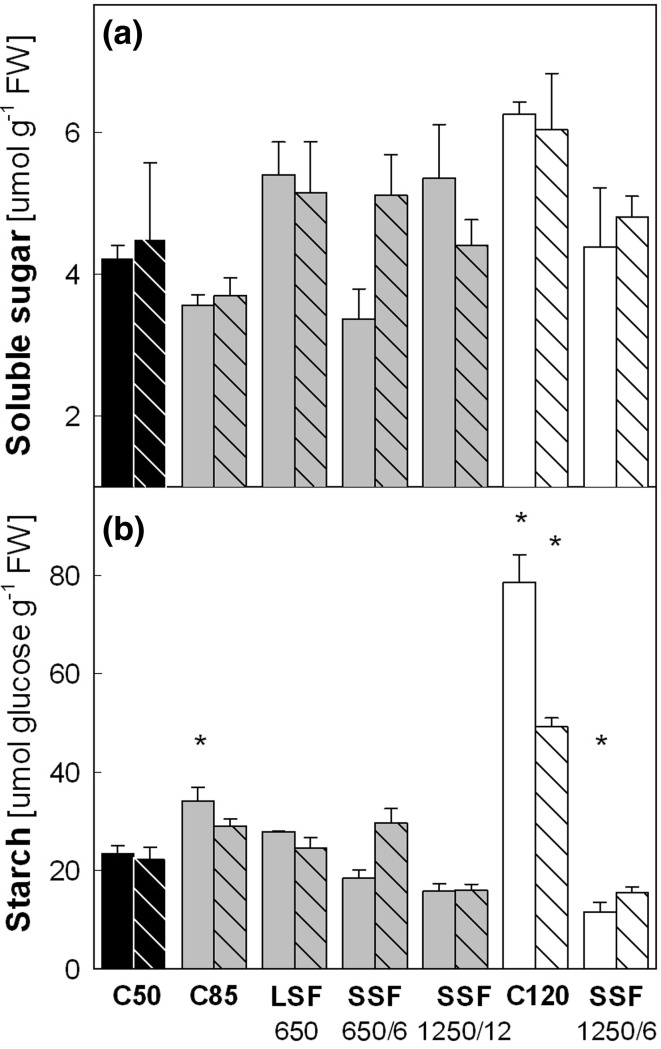
Contents of **a** soluble sugars and **b** starch in leaves of Col-0 plants. **a** Sum of sucrose, glucose and fructose. **b** Starch concentrations measured as glucose. Leaf samples for carbohydrate assay were harvested after 10 h of illumination by different light regimes on day 2 (*solid bars*) and day 5 (*striped bars*). The daily total PAR of different light regimes was ca. 2.1 (*black bars*), 3.6 (*gray bars*) and 5.1 (*white bars*) mol photons m^−2^ day^−1^. *Asterisks* indicate significant differences (*P* < 0.05) compared to the C 50 samples of the same day. Data are means of three plants (±SE)

In order to see whether the observed changes in PSII activity were reflected in the carbohydrate status of these plants, non-structural carbohydrate was analyzed in mature leaves harvested in the evening (after 10 h of illumination by the different light regimes) on day 2 and 5 (Fig. 4). The concentrations of soluble sugars (the sum of glucose, fructose, and sucrose) varied in leaves under the different light regimes (Fig. 4a), yet the differences between C 50 and other treatments were not significant. Higher starch levels were found in C 85 and C 120 on day 2 (Fig. 4b); especially, the leaf starch content in C 120 was more than three times that of C 50. The starch levels then declined in both C 85 and C 120 by day 5 although the plants in C 120 still had twice as much starch as in C 50. None of these changes in starch was accompanied by similar changes in soluble sugar (Fig. 4a). The LSF 650 treatment, which was applied daily at around midday, did not obviously affect the leaf starch content in the evening. The SSF treatments tended to impair starch accumulation with the largest and significant decrease found in SSF 1250/6 on day 2. Leaf starch contents recovered in SSF 650/6 by day 5, but not in SSF 1250/12 and SSF 1250/6. Again, the changes in soluble sugar did not parallel the changes in starch, except for their tendency to recover together in SSF 650/6.

### Leaf growth under different sunfleck conditions

**Fig. 5:**
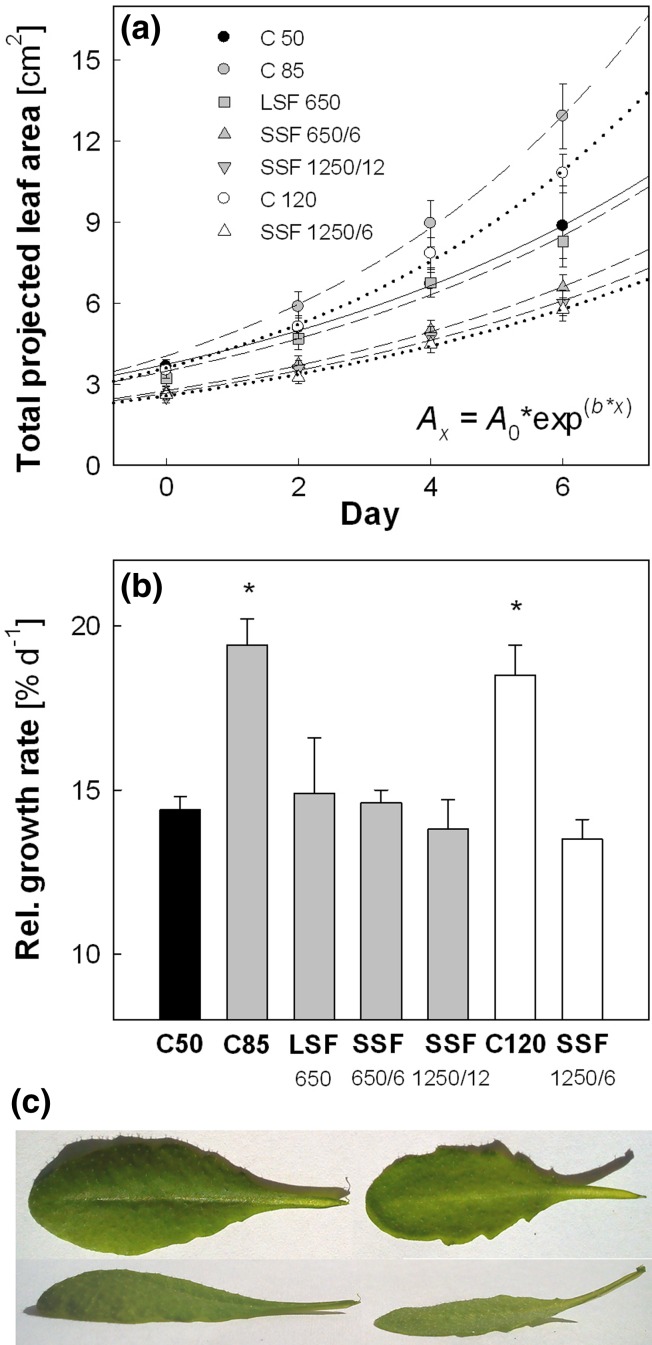
Response of leaf growth in Col-0 plants to different light regimes. **a** Development of the projected total leaf area. Data of each treatment were fitted to an exponential growth function (*r* ^*2*^ > 0.96 for all data sets) to obtain mean relative growth rates. **b** Relative growth rates ( % day^−1^). The daily total PAR of different light regimes was ca. 2.1 (*black symbols* and *bar*), 3.6 (*gray symbols* and *bars*) and 5.1 (*white symbols* and *bars*) mol photons m^−2^ day^−1^. *Asterisks* in **b** indicate significant differences (*P* < 0.05) compared to C 50. Data are means of 20 plants (±SE). **c** A top-view and a side-view of leaves grown in C 50 (*left*) or SSF 1250/6 (*right*)

Leaf area development was monitored by measuring the projected total leaf area of individual Col-0 plants during the 7-day light treatments (Fig. 5a). All data were fitted to an exponential growth function (Eq. 6) to calculate the mean RGR (% day^−1^). In this experiment, the plants had an initial projected total leaf area of ca. 3 cm^2^ on day 0. Figure 5b summarizes the mean RGR values of the plants in the different light regimes. Compared with the RGR of about 14.5 % day^−1^ in C 50, the values in C 85 and C 120 were equally higher (18.5~19.5 % day^−1^). Neither LSF nor SSF significantly altered leaf RGR, although the values tended to decline in SSF 1250/12 and SSF 1250/6; the RGR found in SSF 1250/6 (13.5 % day^−1^) corresponded to 93 % of C 50. We noticed that all plants developed flat leaf lamina under SSF, instead of dome-shaped lamina found in C 50 (Fig. 5c). Since the area of a dome-shaped leaf is larger than the area of its projection, our growth analysis method based on projected leaf area underestimates the area of dome-shaped leaves, but not flat leaves. Consequently, the calculated values of SSF-induced decline in leaf RGR are probably underestimation.

### Responses of different *Arabidopsis* accessions to SSF

**Fig. 6:**
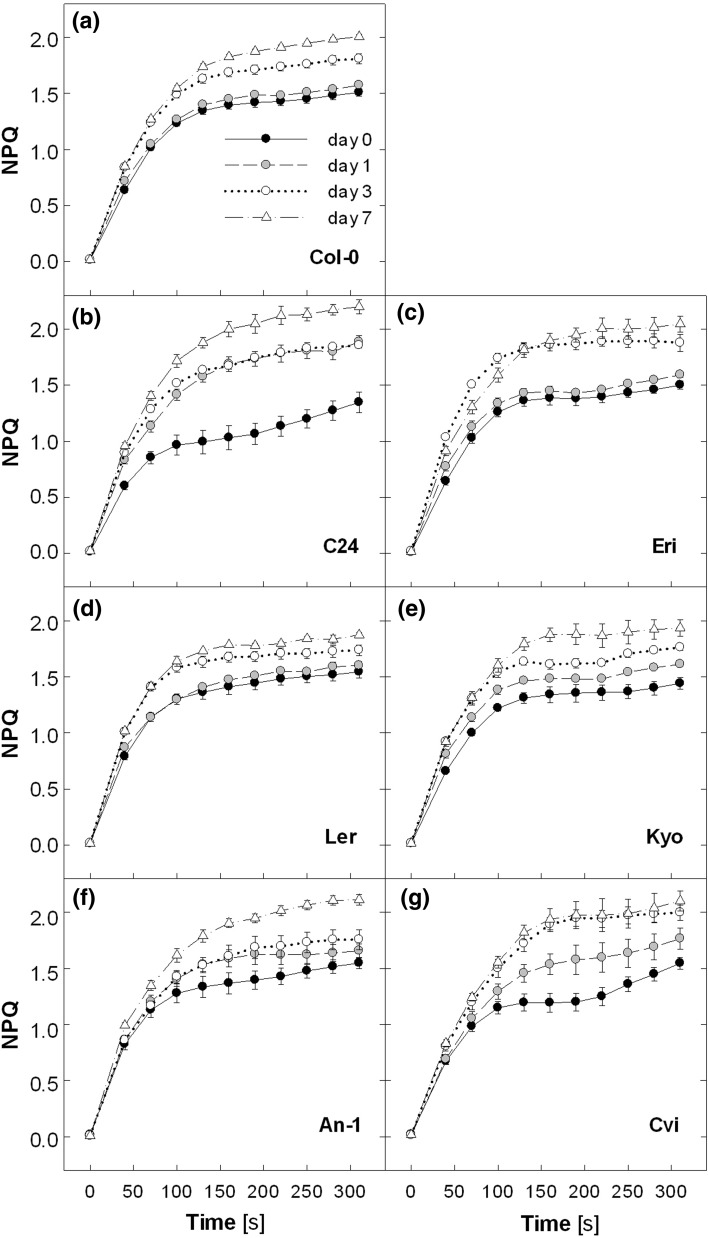
Non-photochemical quenching (NPQ) measured in leaves of different *Arabidopsis* accessions during 7-day exposure to SSF 1250/6. The NPQ was induced by illumination at 1,000 μmol photons m^−2^ s^−1^ for 5 min. The maximal PSII efficiency of dark-adapted leaves at the beginning of the measurements was 0.78–0.82 for all plants during the 7-day experiment. Data are means of 10~12 plants for Col-0 and 3~4 plants for other accessions (±SE)

Having seen the strong upregulation of NPQ by SSF in Col-0 plants, we asked whether different *Arabidopsis* accessions respond to SSF in the same manner. A second series of experiments was conducted by means of six accessions (C24, Eri, L*er*, Kyo, An-1, and Cvi) in addition to Col-0 and exposing them to SSF 1250/6, the sunfleck treatment with both higher intensity and frequency to compare genotypic differences in SSF responses. All accessions uniformly upregulated the NPQ capacity in SSF 1250/6 (Fig. 6). The response of Col-0 plants (Fig. 6a) was essentially the same as in the first experiment (Fig. 1g). The highest NPQ of 2.2 (±0.06 SE) was found in C24 on day 7 (Fig. 6b).

**Fig. 7:**
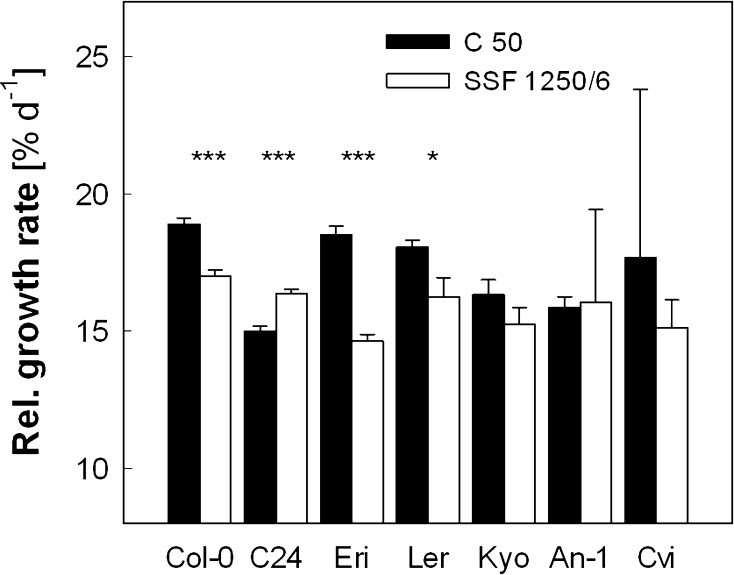
Response of leaf growth to SSF 1250/6 in different *Arabidopsis* accessions. Relative growth rate was obtained by fitting the data of the projected total leaf area to an exponential function (*r* ^*2*^ > 0.98 for all data sets), as illustrated in Fig. 5a. *Asterisks* indicate significant differences (***P < 0.001; *P < 0.05) between C 50 and SSF 1250/6 for each accession. Data are means of 11~15 plants (±SE)

In contrast to the uniform increase in NPQ (Fig. 6), the response of leaf RGR differed among the accessions (Fig. 7). The plants had the following initial projected total leaf area (in cm^2^) on day 0 (*n* = 11–15, ±SE): Col-0, 2.1 ± 0.1; C24, 3.7 ± 0.2; Eri, 3.5 ± 0.4; L*er*, 2.1 ± 0.2; Kyo, 3.2 ± 0.4; An-1, 3.4 ± 0.3; and Cvi, 3.0 ± 0.2. The initial leaf area of Col-0 plants was ca. 30 % smaller in this experiment than in the first experiment (3 cm^2^, Fig. 5a), presumably due to the stratification introduced in the second experiment. The average leaf RGR of about 19 % day^−1^ was measured in Col-0 under C 50 (Fig. 7), which is much higher than in the first experiment (14.5 % day^−1^, Fig. 5b). As expected, the treatment with SSF 1250/6 decreased the leaf RGR in Col-0 (−10 %), Eri (−21 %) and L*er* (−10 %) compared with the values under C 50; the small decrease found in Kyo was not statistically significant. On the contrary, SSF 1250/6 resulted in an increase in leaf RGR in C24 (+9 %), which had the lowest RGR under the C 50 condition. The leaf growth analysis in An-1 (SSF 1250/6) and Cvi (C 50) was hampered by large variability among individual plants. As observed in Col-0 in the first experiment (Fig. 5c), leaf morphology was changed in all accessions during the 7-day exposure to SSF 1250/6 from dome-shaped lamina in C 50 to flat lamina in SSF 1250/6 (data not shown).

### Photoprotective responses to SSF in different *Arabidopsis* accessions

The NPQ measurements (Fig. 6) indicated conserved photoprotective responses to SSF among the seven accessions, while the leaf growth analysis revealed some divergence. To ascertain whether the SSF-induced upregulation of NPQ involved similar photoprotective mechanisms in different accessions, photosynthetic pigment composition was analyzed in mature leaves on day 0 and 7. Three accessions, Col-0, C24, and Eri, were chosen for the analysis because they exhibited distinct responses of leaf RGR (Fig. 7): a moderate decrease (Col-0), a strong decrease (Eri, Northern European accession), and an increase (C24, Southern European accession) in SSF 1250/6.

**Fig. 8:**
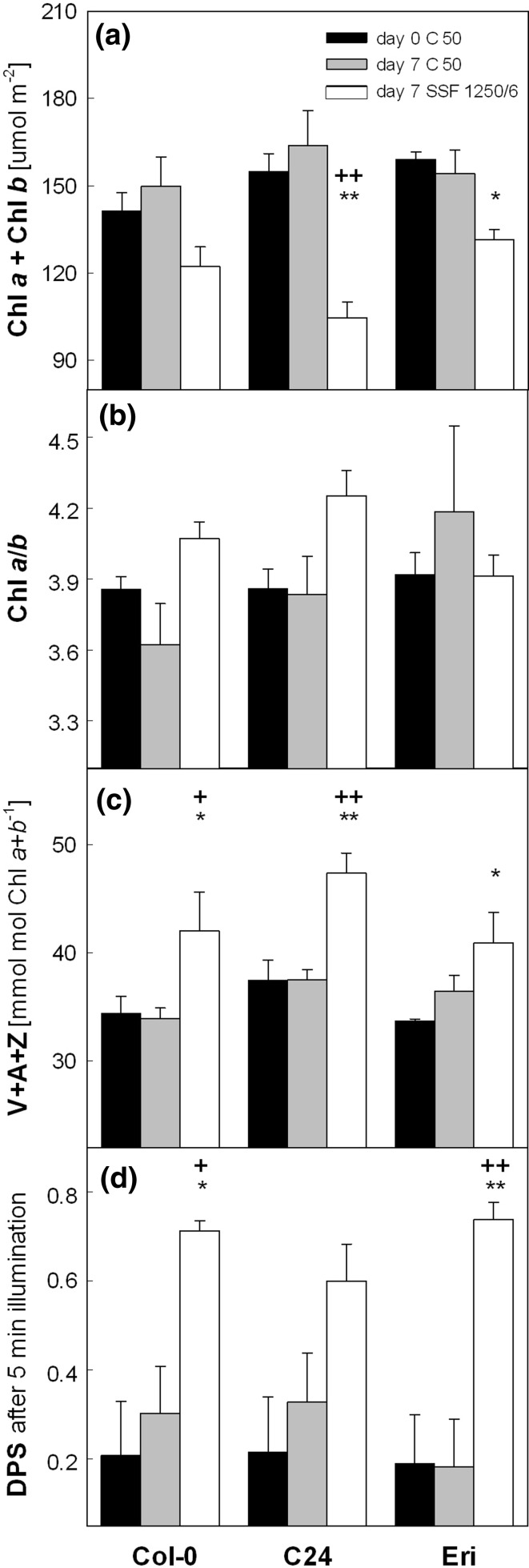
Changes in leaf pigment composition of Col-0, C24 and Eri. **a** Total chlorophyll content. **b** Chlorophyll *a* to chlorophyll *b* ratio. **c** Pool size of the xanthophyll-cycle pigments. Leaf samples for **a**–**c** were harvested at the end of the night period on day 0 (all plants under C 50) and day 7 (C 50 or SSF 1250/6). None of the leaves contained A or Z except a single SSF sample of Col-0 in which a small amount of A was detected on day 7. **d** De-epoxidation state (DPS) of the xanthophyll-cycle pigments after 5-min exposure to 1,000 μmol photons m^−2^ s^−1^. The DPS was calculated as (A + Z)/(V + A + Z). For each accession, *asterisks* indicate significant differences (**P < 0.01; *P < 0.05) between day 0 (C 50) and day 7 of SSF 1250/6; plus signs indicate significant differences (^++^ *P* < 0.01; ^+^ *P* < 0.05) between C 50 and SSF 1250/6 on day 7. Data are means of 3~4 plants (±SE)

In the C50 condition, dark-adapted plants (sampled at the end of the night) of the three accessions were comparable in terms of leaf Chl content (Fig. 8a), Chl *a* to Chl *b* ratio (Chl *a*/*b*; Fig. 8b) and pool size of the xanthophyll-cycle pigments V, A and Z (V + A + Z; Fig. 8c). A 5-min exposure of the dark-adapted plants to ca. 1,000 μmol photons m^−2^ s^−1^ (as was applied for the measurements of the maximal NPQ in Fig. 6) strongly increased the de-epoxidation state of the xanthophyll-cycle pigments (DPS = (A + Z)/(V + A + Z); Fig. 8d) in all plants. These pigment parameters change in leaves of a variety of species during HL acclimation (Demmig-Adams and Adams 1992; Matsubara et al. 2009), including *Arabidopsis* (Ballottari et al. 2007; Kalituho et al. 2007), or tropical rainforest plants under sunfleck/gap conditions (Logan et al. 1997; Watling et al. 1997b; Adams et al. 1999; Krause et al. 2001).

The SSF 1250/6 treatment decreased the Chl content in all three accessions (Fig. 8a), which was accompanied by somewhat increased Chl *a*/*b* for Col-0 and C24, but not for Eri (Fig. 8b). The levels of V + A + Z relative to Chl increased by 20, 27, and 17 % in Col-0, C24, and Eri, respectively (Fig. 8c). The concentrations of other carotenoids (β-carotene, lutein, and neoxanthin) were similar in the three accessions and did not change significantly in SSF 1250/6 by day 7 (data not shown). Neither A nor Z was detected in leaves at the end of the night period, except a single Col-0 leaf in SSF 1250/6 that had some A (12 mmol mol Chl^−1^, data not shown). Brief exposure to HL quickly induced 60~70 % conversion of V to A and Z in the SSF 1250/6 plants, while the same HL exposure resulted in much less de-epoxidation (20~30 %) in the C 50 plants (Fig. 8d).

**Fig. 9:**
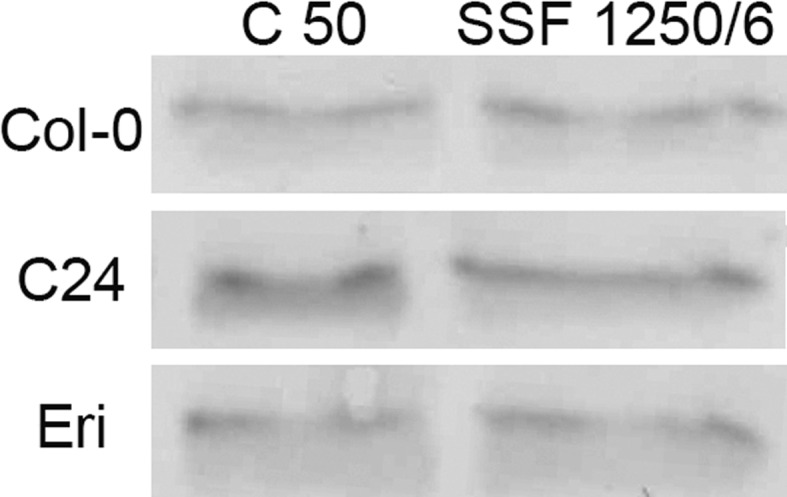
Immunoblot analysis showing PsbS protein levels in mature leaves of Col-0, C24 and Eri acclimated to the C50 or SSF 1250/6 conditions. Extracts from three replicate leaves (from three replicate plants) were harvested on day 7 and pooled for each genotype and treatment

Light-induced formation of NPQ is triggered by a pH decrease in the thylakoid lumen, leading to activation of V de-epoxidase (to form Z) and protonation of the PsbS protein, another essential component of NPQ in higher plants (Li et al. 2000, 2004; Dominici et al. 2002). Independent of the changes in V + A + Z, the amount of the PsbS protein relative to Chl increased in SSF 1250/6 (Fig. 9). The following changes in PsbS levels were found in the three accessions after 7 days of acclimation to SSF 1250/6: +25 % in Col-0, +20 % in C24 and +15 % in Eri.

**Fig. 10:**
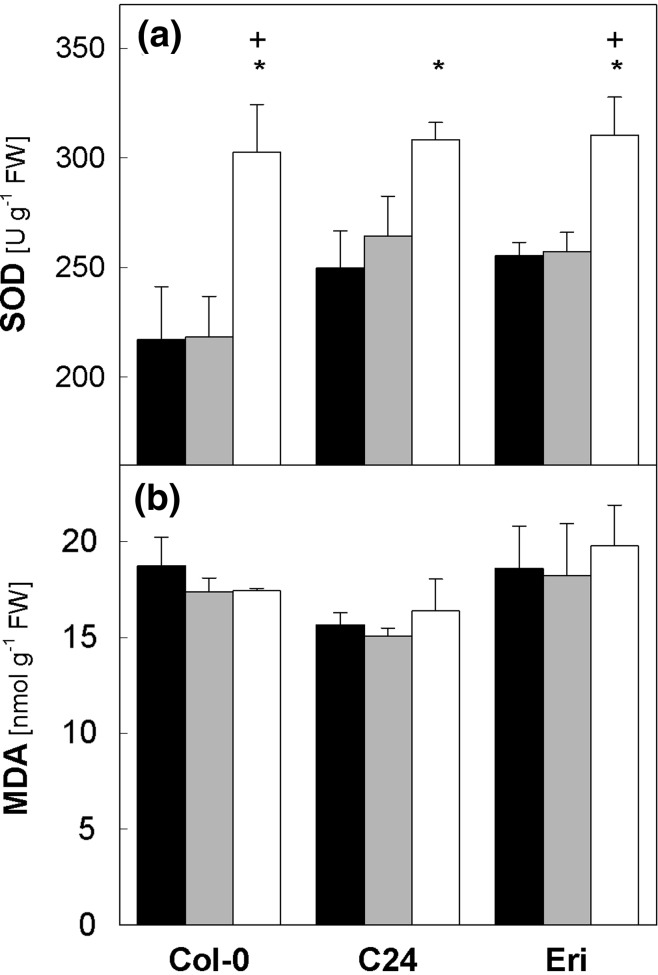
Superoxide dismutase activity (**a**) and malondialdehyde content (**b**) in leaves of Col-0, C24 and Eri. Leaf samples were harvested on day 0 (*black bars*, all plants under C 50) and day 7 (*gray bars*, C 50; *white bars*, SSF 1250/6). For each accession, *asterisks* indicate significant differences (*P* < 0.05) between day 0 (C 50) and day 7 of SSF 1250/6; *plus signs* indicate significant differences (*P* < 0.05) between C 50 and SSF 1250/6 on day 7. Data are means of four plants (±SE)

The enzyme SOD catalyzes disproportionation of O_2_^−^ to H_2_O_2_ and O_2_. In chloroplasts, it acts as the first enzyme in the water–water cycle which allows linear electron transport without ATP consumption (Osmond and Grace 1995; Asada 1999), thus contributing to acidification of the thylakoid lumen needed for rapid induction of NPQ and activation of V de-epoxidase. The leaf SOD activity was somewhat lower in Col-0 than in C24 and Eri when these plants were under C 50 (Fig. 10a). The SSF 1250/6 treatment induced marked upregulation of SOD activity in all three accessions, resulting in similarly high values on day 7. The MDA levels found in mature leaves at the end of the night period did not differ under the two light regimes (Fig. 10b), which is in line with the high *F*_v_/*F*_m_ measured in SSF 1250/6 (see legend to Figs. 1 and 6).

Table 1 summarizes the results of two-way ANOVA analyzing the effects of accessions (Col-0, C24, and Eri) and light treatments (C 50 and SSF 1250/6) on the changes of the parameters described above. The leaf RGR is the only trait for which interaction between the effects of accessions and treatments was found. Genotypes and treatments seem to independently influence the maximal NPQ levels, whereas variations in the Chl content, V + A + Z, DPS, and SOD activity can be explained by the light treatments alone.

**Table 1:** Summary of two-way ANOVA test

	Leaf RGR	Max NPQ	Chl *a* + *b*	Chl *a*/*b*	V + A + Z	DPS	SOD	MDA
Accessions	(0.009*)	*<0.001*	0.594	0.562	0.067	0.743	0.234	0.228
Treatments	(0.208*)	*<0.001*	*<0.001*	0.258	*<0.001*	*<0.0011*	*<0.0011*	0.538
Interaction (accessions × treatments)	*<0.001*	0.694	0.103	0.185	0.378	0.400	0.437	0.915

Effects of accessions (Col-0. C24 and Eri) and treatments (C 50 and SSF 1250/6) on different parameters were tested. Shown are *P* values for each set of test. Significant effects are marked italics

* Due to significant interactions between accessions and treatments, the main effect of each factor cannot be properly determined

## Discussion

### Acclimation to fluctuating light environment: effects of light intensity, duration, and frequency

**Fig. 11:**
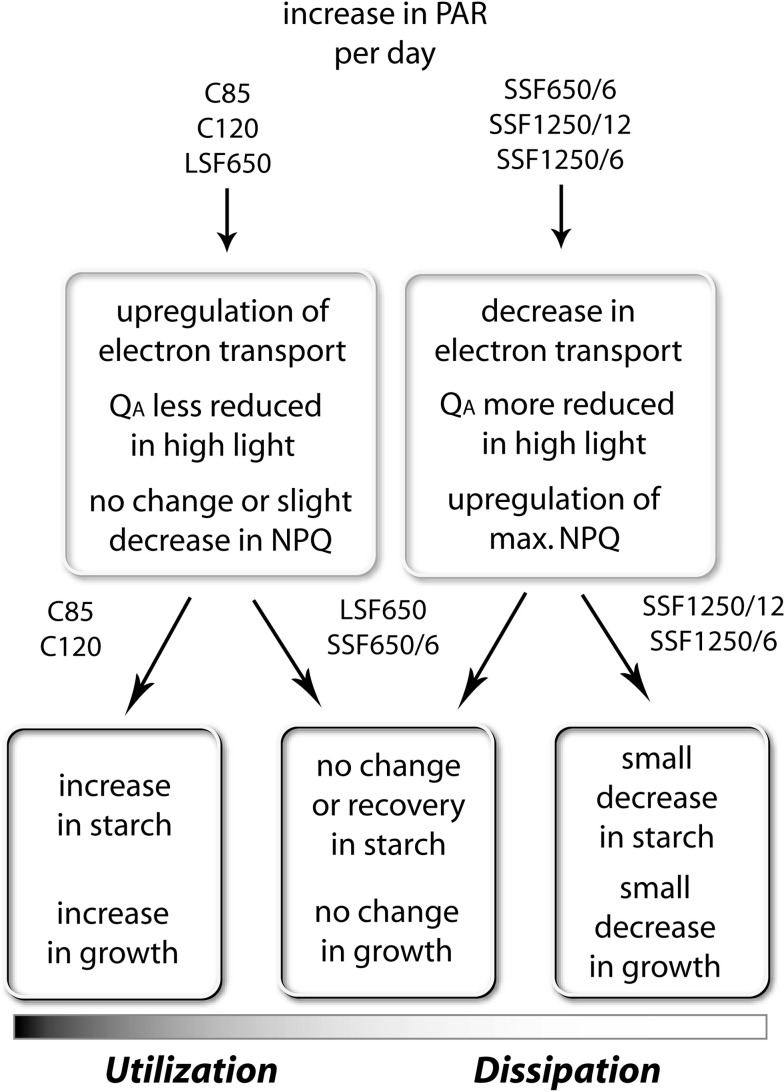
A diagram summarizing the responses of *Arabidopsis* (Col-0) during 7-day acclimation to constant (C 85, C 120) or fluctuating light environment with long (LSF 650) or short sunflecks (SSF 650/6, SSF 1250/12, SSF 1250/6). All plants were acclimated to the C 50 condition before starting the experiments on day 0

Figure 11 gives an outline of the responses of Col-0 during acclimation to different light regimes. The 7-day treatments were long enough to study these acclimatory changes in *Arabidopsis* plants. The NPQ capacity increased in mature leaves of the SSF plants in which Q_A_ was more strongly reduced upon HL exposure (Figs. 1 and 2); as 1-qp decreased on day 7 to reach a level as low as in C 85 and LSF 650 (SSF 650/6) or to restore the initial level on day 0 (SSF 1250/12, SSF 1250/6), deceleration of NPQ upregulation was observed. Likewise, the NPQ capacity in C 85, C 120, and LSF 650 did not change, or even declined slightly (Fig. 1), as the capacity for Q_A_ oxidation and electron transport increased in these plants (Figs. 2 and 3). These results underline opposite and complementary responses of NPQ and electron transport under the different light conditions used in this study (Fig. 11, *upper boxes*).

Our data in SSF 650/6 clearly show that NPQ enhancement precedes upregulation of electron transport during acclimation to SSF (Figs. 1d, 2d, and 3d) presumably to cope with an acute threat of photo-oxidation. Since both SSF 1250/12 and SSF 1250/6 increased the maximal NPQ and suppressed the upregulation of Q_A_ oxidation and electron transport almost equally and more strongly than SSF 650/6 (Figs. 1–3), it seems that the intensity of SSF has a great impact on these acclimatory responses in *Arabidopsis* plants.

How about the duration and the frequency of sunflecks? The two treatments SSF 650/6 and LSF 650 revealed distinct initial effects of the sunflecks with contrasting duration and frequency (but the same intensity): upregulation of NPQ and photoprotection in SSF 650/6 and upregulation of Q_A_ oxidation and electron transport in LSF 650 (Fig. 11). In a previous study (Yin and Johnson 2000), 7-day exposure of *Arabidopsis* plants (L*er*) grown under 100 μmol photons m^−2^ s^−1^ to a light cycle of 3 h/3 h between 100 and 475 μmol photons m^−2^ s^−1^ during the daytime caused a >70 % increase in the maximal leaf oxygen evolution, while shorter cycles (15 min/15 min and 1 h/1 h) hardly had an effect; when PAR fluctuated between 100 and 810 μmol photons m^−2^ s^−1^, the shortest cycle of 15 min/15 min was most efficient in enhancing the oxygen evolution capacity. Although the light regimes used by Yin and Johnson (2000) are quite different from our sunfleck treatments, it is plausible that the reduction in 1-qp (Fig. 2c) and the increase in ETR (Fig. 3c) found in LSF 650 reflects, at least in part, the acclimatory enhancement of PSII activity described in that study. Notably, a single 12-h exposure to C 85 or C 120, or a daily 40-min exposure to LSF 650 for a couple of days was enough to bring about small but significant initial changes in 1-qp and ETR (Figs. 2c and 3c), demonstrating the ability of *Arabidopsis* plants to rapidly increase the capacity for photosynthetic electron transport.

Unlike in C 85 and C 120, however, the increased electron transport in LSF 650 did not lead to higher starch accumulation or enhanced leaf expansion (Fig. 11, *lower boxes*). The 40-min exposure to LSF, which raised the leaf temperature from 21~22 to 27~28 °C, may have promoted photorespiration (if the treatment decreased the stomatal conductance) and/or mitochondrial respiration, including rapid upregulation of alternative oxidase (Osmond and Grace 1995; Leakey et al. 2004; Yoshida et al. 2011). Also, additional carbon fixed during LSF may have been transported out of the mature leaves to support sink organs such as growing roots, as was found in *Nicotiana tabacum* upon PAR increase from 60 to 300 μmol photons m^−2^ s^−1^ (Nagel et al. 2006).

Together, these results, showing distinct acclimatory responses of Col-0 plants to constant light, LSF, and SSF, strongly suggest the involvement of light intensity, duration, and frequency in adjusting photoprotection and carbon gain at different levels (Fig. 11). Plant acclimation entails activation/deactivation and upregulation/downregulation of various physiological processes, including restructuring and reorganization of relevant components. In addition to the intensity and acuteness of the signal, factors such as how quickly each of these processes can react (response time) and how long certain signals can last in the cell probably gain importance for determining the acclimatory response to fluctuating conditions. Building on the knowledge provided by the numerous studies on acclimation to (constant or less dynamic) HL and LL, future investigations could elucidate the roles of different processes and signals associated with regulation of photosynthetic acclimation, e.g., plastoquinone and stromal redox state, ATP/ADP ratio, sugars, and ROS (Pfannschmidt 2003; Walters 2005), in fluctuating light environment.

### Photoprotective acclimation to SSF is conserved among different *Arabidopsis* accessions

Carbon gain during sunflecks can constitute a substantial fraction of the daily total carbon gain for leaves and plants in shaded environments (Pearcy and Calkin 1983). Yet, utilization of sunflecks is restricted by photosynthetic induction, especially by limited regeneration of ribulose-1,5-bisphosphate in the first minutes (Chazdon and Pearcy 1986a; Pons et al. 1992). During LL periods, the photosynthetic induction state is lost more quickly in fast-growing sun plants than in shade-tolerant understorey plants (Chazdon and Pearcy 1986a; Pons et al. 1992) although the initial rate of decrease can be comparable in different species of contrasting habitats (approx. −30 % in the first 5 min; Ögren and Sundin 1996).

Consistent with such a limitation to utilize SSF for photosynthesis, we found lower ETR (Fig. 3), unchanged or slightly reduced carbohydrate accumulation (Fig. 4) and leaf expansion (Fig. 5) in Col-0 plants under the SSF conditions compared with C 50, despite the much higher (+70 % or +140 %) daily total irradiance. Because *Arabidopsis* is a typical open-field plant, the ability to utilize sunflecks may not be as vital as for forest understorey species. Instead, a major acclimatory response of *Arabidopsis* to SSF is characterized by the upregulation of the NPQ capacity (Fig. 1). The maximal NPQ levels rapidly increased in all plants during the SSF treatments, which also resulted in faster light-induced NPQ formation, as indicated by the higher values already after 30 s in HL.

While species may vary in their photosynthetic responses to sunflecks (Chazdon and Pearcy 1986b; Ögren and Sundin 1996; Watling et al. 1997a), SSF 1250/6 induced uniform upregulation of NPQ in all *Arabidopsis* accessions examined in the present study (Fig. 6). The analysis of photosynthetic pigments in Col-0, C24, and Eri (Fig. 8) further corroborates the highly conserved photoprotective responses in these plants. While the variations in the biochemical traits are mainly attributable to acclimation to light environment, the maximal NPQ level seems to be determined environmentally as well as genetically (Table 1). This is in agreement with the finding in *Arabidopsis* by Jung and Niyogi (2009), namely the presence of two quantitative trait loci (QTL) for high NPQ (*HQE1* and *HQE2*) and a poor correlation between intraspecific NPQ variations and the biochemical traits associated with NPQ.

Reduction in leaf Chl content (Fig. 8a) is a typical symptom of HL acclimation in a wide range of species (e.g., Demmig-Adams and Adams 1992; Matsubara et al. 2009). When grown under constant HL, *Arabidopsis* plants accumulate less Chl but more PSII having smaller light-harvesting antennae compared to the plants in LL (Bailey et al. 2001; Ballottari et al. 2007; Kalituho et al. 2007), which results in higher Chl *a*/*b*. This tendency was observed in two out of the three accessions under SSF 1250/6 (Fig. 8b), whereas the V + A + Z amount relative to Chl increased invariably in all three accessions (Fig. 8c). These changes in V + A + Z are not a mere consequence of Chl degradation because V + A + Z did not increase when the Chl content decreased in SSF 650/6 (data not shown). Instead, the synthesis of V + A + Z must have been upregulated in leaves during acclimation to SSF 1250/6.

The increase in V + A + Z was accompanied by faster de-epoxidation of V to A and Z upon HL exposure (Fig. 8d). An extra pool of V filling the peripheral xanthophyll biding sites (site V1) of the major light-harvesting antenna complexes of PSII (Caffarri et al. 2001) may have provided quickly available substrates for V de-epoxidase to allow rapid formation of Z, which is an essential component of NPQ (Demmig-Adams 1990; Niyogi et al. 1998) and can also act as antioxidant to protect thylakoid membranes against lipid peroxidation (Fig. 10; Havaux and Niyogi 1999; Havaux et al. 2007). In addition, higher levels of the PsbS protein (relative to Chl) found in SSF 1250/6 (Fig. 9) could also enhance NPQ formation. The fact that the lack of PsbS in *Arabidopsis* *npq4* mutants is not disadvantageous in constant PAR but reduces fitness under fluctuating light conditions (Külheim et al. 2000) is also in line with the NPQ upregulation found in all SSF plants in the present study (Figs. 1 and 6). Combined with adjustment of other mechanisms, e.g., marked upregulation of the SOD activity (Figure 10a; Grace and Logan 1996; Abarca et al. 2001), these changes to reorganize pigment–protein complexes and enhance photoprotective/antioxidative capacities enable LL-grown *Arabidopsis* plants to acclimate to SSF conditions without extensive photoinhibition and lipid peroxidation (Fig. 10b).

## Conclusions

Fluctuations in PAR, with different combinations of duration, frequency, and intensity, elicit various acclimatory responses in plants. In *Arabidopsis*, brief and strong increase in PAR generally enhances photoprotection and energy dissipation, presumably because they are unable to quickly utilize the additional light energy provided in this form. Longer periods of high PAR seem to allow upregulation of electron transport rather than NPQ. In conjunction with the use of different genotypes, experiments with fluctuating light regimes will promote our understanding of the regulatory mechanisms in plant acclimation to light environment.

## Abbreviations

A: Antheraxanthin
C: Constant light
Chl: Chlorophyll
Chl *a*/*b*: Chlorophyll *a* to chlorophyll *b* ratio
DPS: De-epoxidation state of the xanthophyll-cycle pigments
ETR: Relative electron transport rate of photosystem II
*F*_v_/*F*_m_: Maximal photosystem II efficiency in a dark-adapted state
FW: Fresh weight
HL: High light
LL: Low light
LSF: Long sunflecks
MDA: Malondialdehyde
NPQ: Non-photochemical quenching
PAR: Photosynthetically active radiation
PSII: Photosystem II
1-qp: Reduction state of the bound primary quinone Q_A_ in photosystem II
RGR: Relative growth rate
SOD: Superoxide dismutase
SSF: Short sunflecks
V: Violaxanthin
Z: Zeaxanthin
